# Story of 20 Years of Triumph: A Case Report of Two Patients With Stage IV Granulosa Cell Tumor of the Ovary

**DOI:** 10.7759/cureus.57615

**Published:** 2024-04-04

**Authors:** Sameen Bin Naeem, Maryam Imran, Mansoor Abbas, Muhammad Awais Majeed, Muhammad Ahsan Jamil, Mahnoor Samreen, Neelam Siddiqui

**Affiliations:** 1 Medical Oncology, Shaukat Khanum Memorial Cancer Hospital and Research Centre, Lahore, PAK; 2 Oncology, Shaukat Khanum Memorial Cancer Hospital and Research Centre, Lahore, PAK; 3 Radiology, Shaukat Khanum Memorial Cancer Hospital and Research Centre, Lahore, PAK

**Keywords:** ovarian cancer, letrozole, tamoxifen, postmenopausal woman, ovary cancer, granulosa cell tumour

## Abstract

Ovarian granulosa cell tumors (GCTs) are rare neoplasms with a unique incidence pattern peaking in postmenopausal women. This case report presents two instances of stage 4 recurrent adult GCTs with a prolonged 20-year follow-up. Patient 1, diagnosed at 54 years, experienced multiple recurrences managed through surgery, hormonal therapy, and chemotherapy, culminating in hepatocellular carcinoma. Patient 2, diagnosed at 67 years, underwent various treatments, including surgery, chemotherapy, and hormonal therapy, demonstrating disease stability. Despite the generally favorable prognosis, these cases highlight the challenges of managing recurrent GCTs, emphasizing the need for tailored therapeutic approaches.

## Introduction

Ovarian cancer is the most common gynecological cancer in Pakistan; however, it is the second most common gynecological cancer in the USA and the Western world [1]. Around 90% of tumors of the ovary are epithelial in origin and primarily affect postmenopausal women [2]. Ovarian sex cord-stromal tumors make up about 7-10% of all primary malignant ovarian tumors, are uncommon, and normally develop in the first two to three decades of life. As an exception, adult granulosa cell tumors (GCTs) of the ovary have a late beginning, peak incidence between 50 and 55 years of age, and only make up 2-5% of all ovarian malignancies [3].

Ovarian GCT has a good prognosis and a propensity for late relapses and can be divided into adult (95%) or juvenile (5%) based on histological findings. Tumorigenesis of adult GCT is driven by forkhead box L2 (FOXL2) mutation, expression of Sma and Mad-related proteins, transforming growth factor-β, and telomerase mutation of reverse transcriptase (TERT) promoter, while juvenile GCT is driven by serine/threonine kinase (AK T1) gene activation [4]. FOXL2 mutation is also sensitive and specific for the diagnosis of adult GCT with 92% accuracy and FLOX2 mutant adult GCT has a similar overall survival as compared to the age-matched general population [4].

For both primary and recurrent tumors, surgery is the preferred course of action [5]. With stage 1C, poor differentiation, a high mitotic index, and tumor rupture on surgical pathology or with progression of malignancy at presentation despite having been completely removed, adjuvant chemotherapy is recommended [6].

In contrast to epithelial ovarian cancers, late relapses are common in GCTs even after a period of five years with recurrent disease occurrence [6].

We report two cases with multiple relapses throughout their course of treatment and successfully completing 20 years of follow-up with stage 4 recurrent GCT of the ovary.

## Case presentation

Patient 1

**Figure 1:**
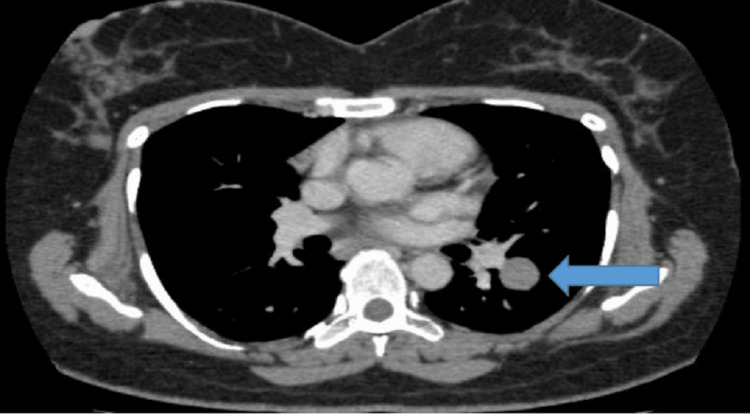
CT of the thorax with contrast (axial view): solitary metastatic deposit of left lower lobe.

A 54-year-old woman known to have hepatitis C, first diagnosed in October 2000 with amenorrhea, underwent left salpingo-oophorectomy, confirmed to have stage 1A disease on histopathology with no residual disease and intact capsule and negative margins. She had her first recurrence in August 2008. She underwent total abdominal hysterectomy and right salpingo-oophorectomy, and histopathology was positive for relapsed disease. She received four cycles of BEP (bleomycin, cisplatin, and etoposide) till November 2008, followed by active surveillance. In 2010, she developed lung metastasis (Figure 1) with no evidence of local residual disease, underwent left lower lobe lobectomy, and histopathology was consistent with recurrent GCT.

**Figure 2:**
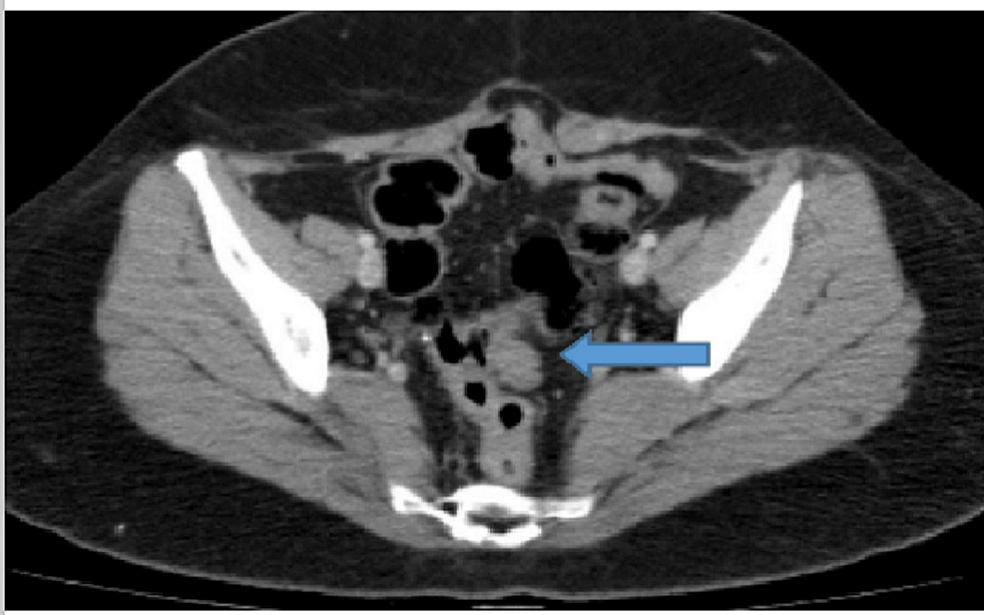
Axial view of CT of the chest, abdomen, and pelvis (CAP) with contrast showing an increase in the size of soft tissue adjacent to the sigmoid colon with an impression of follicles, representing residual ovarian tissue.

She relapsed again in October 2013 with a metastatic lesion abutting the sigmoid colon on a CT scan (Figure 2).

**Figure 3:**
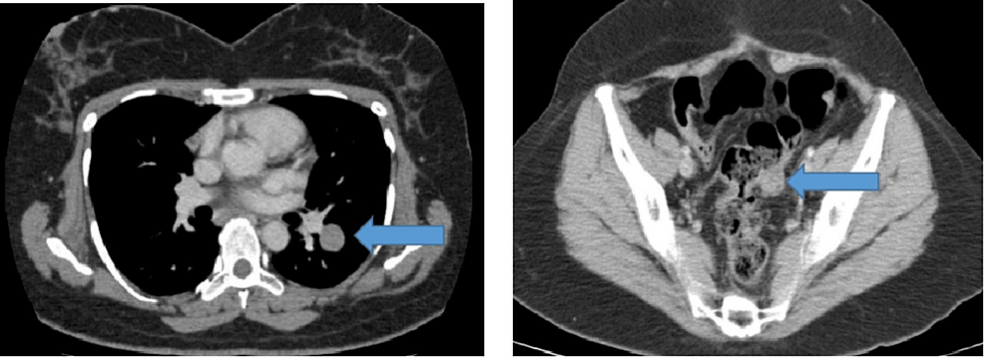
CT of the chest, abdomen, and pelvis (CAP) with contrast (axial views): disease progression by virtue of an increase in the size of left pulmonary and sigmoid colon nodules.

She received hormonal therapy till 2020, tamoxifen for one year, and letrozole for five years, with a stable colonic lesion. She developed progression again in May 2020 with new pulmonary lesions and an increase in the size of the previously stable colonic lesion (Figure 3). She received six cycles of carboplatin (three weekly) and paclitaxel (weekly), followed by 34 cycles of weekly paclitaxel till November 2021, with stable disease on follow-up imaging. The patient wished for a chemotherapy break and was commenced on hormonal therapy (tamoxifen for two weeks alternating with megestrol acetate) to date.

**Figure 4:**
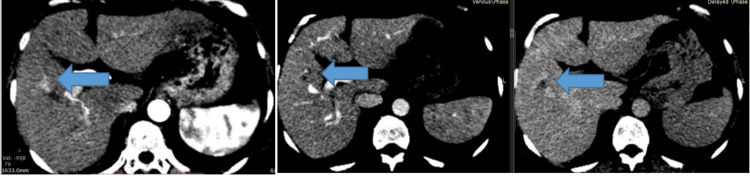
Axial sections of CT triphasic of liver: small area of arterial enhancement in segment 5 with washout on venous and delayed phase concerning for hepatocellular carcinoma.

**Figure 5:**
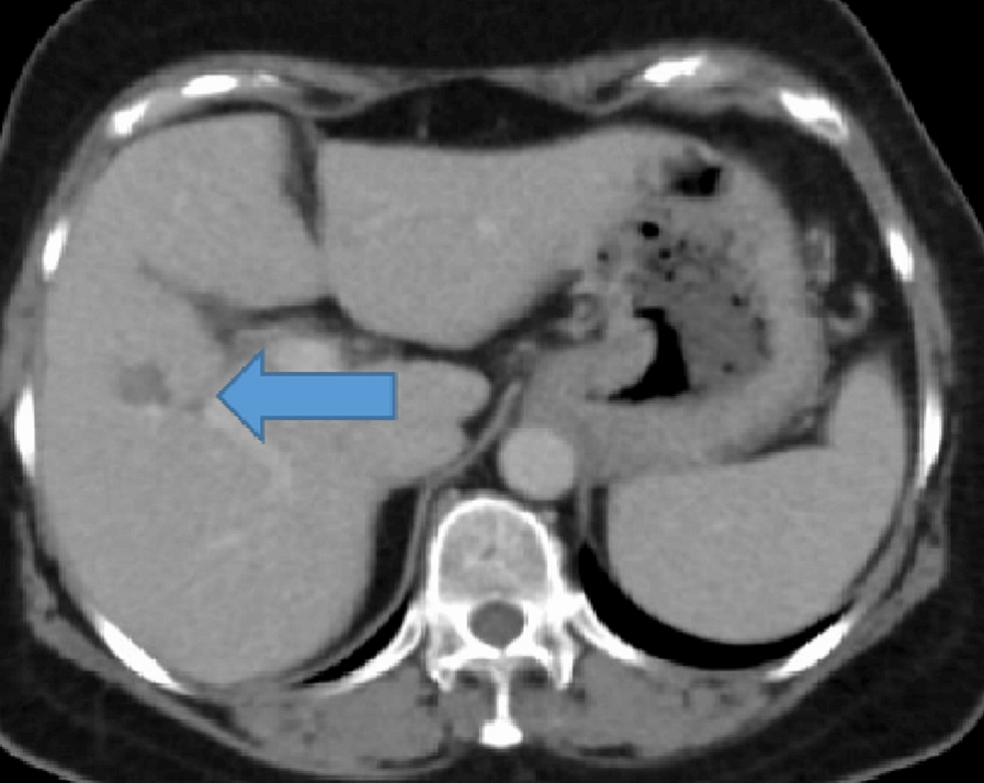
Axial view of CT of the chest, abdomen, and pelvis (CAP) showing satisfactory post-radioablative changes in segment 5 of the liver.

On follow-up in June 2022, her alpha-fetoprotein started rising, and the imaging findings were consistent with hepatocellular carcinoma, a very early stage as per the Barcelona Clinic Liver Cancer (BCLC) criteria (Figure 4). She received radiofrequency ablation of her liver lesion with no residual/recurrent lesions on follow-up imaging (Figure 5).

Patient 2

**Figure 6:**
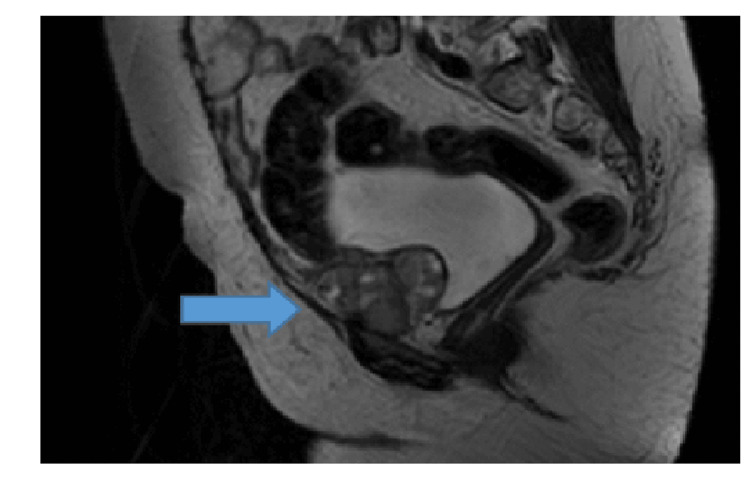
MRI pelvis T2 images showing multilobulated soft tissue masses in the retropubic fat anterior to the urinary bladder. There is no MRI evidence of invasion.

A 67-year-old woman, known to have hypertension, was first diagnosed in December 2004 with stage 1A adult GCT, when she had a total abdominal hysterectomy and right salpingo-oophorectomy from outside hospital for dysfunctional uterine bleeding. She was referred to our center in December 2007 after the excision of a left adnexal mass, consistent with relapsed disease (Figure 6).

**Figure 7:**
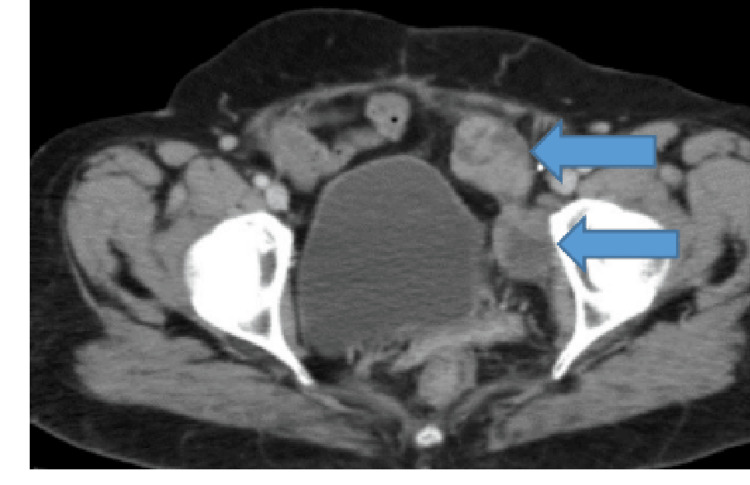
CT scan axial view showing heterogenous masses along the left iliac vessels suggestive of recurrent disease in the left hemi pelvis.

**Figure 8:**
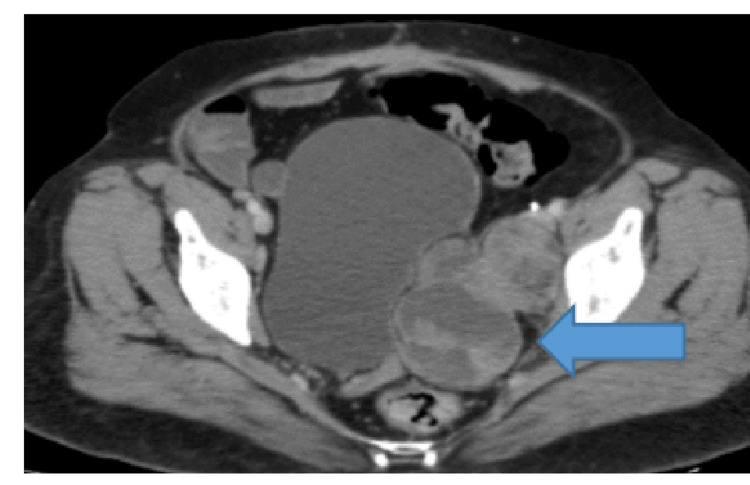
CT scan axial view showing interval increase in size and number of the pelvis side wall masses.

**Figure 9:**
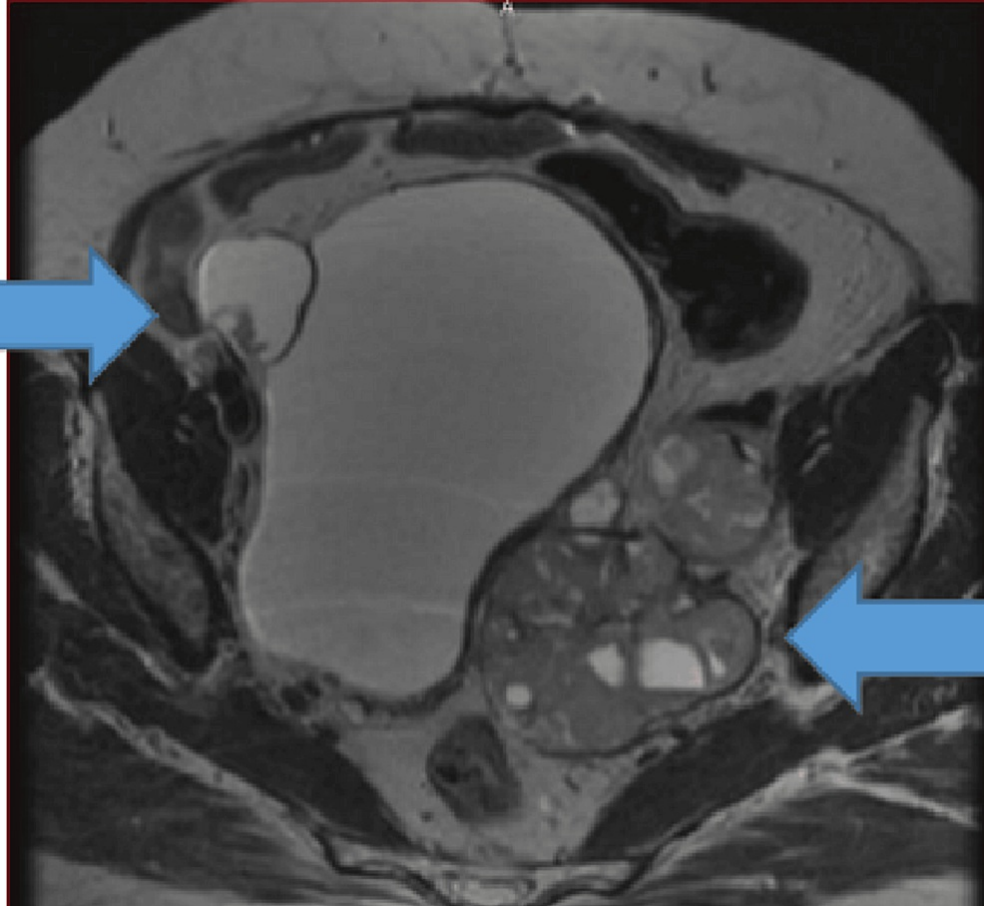
MRI of the pelvis T2 sequence axial view showing interval progression of the large multilobulated left adnexal mass and new right adnexal complex masses, suggesting disease progression.

She received six cycles of carboplatin/cyclophosphamide. In October 2008, she developed a recurrence with a tumor invading the urinary bladder. She underwent resection of the pelvic mass with incomplete cytoreduction, followed by four cycles of BEP. She relapsed again in March 2013 with a left iliac fossa mass (Figure 7) and received six cycles of carboplatin/paclitaxel, followed by pelvic mass resection. She relapsed for the 4th time in August 2016 with pelvic side wall masses (Figure 8) unresectable and was treated with six cycles of carboplatin/paclitaxel. In August 2018, her disease progressed with an increase in adnexal masses and iliac nodes (Figure 9).

**Figure 10:**
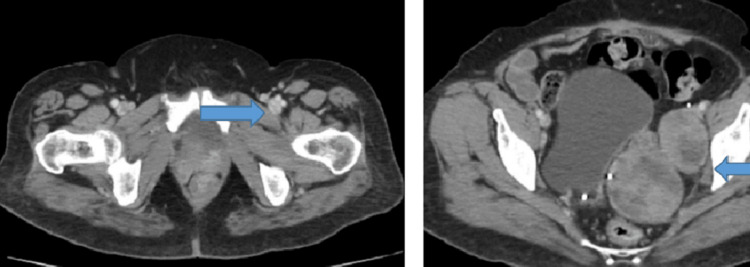
CT scan axial views showing an interval increase in the size of complex lobulated predominantly solid left adnexal mass inseparable from the pelvic side wall and right external iliac lymphadenopathy.

**Figure 11:**
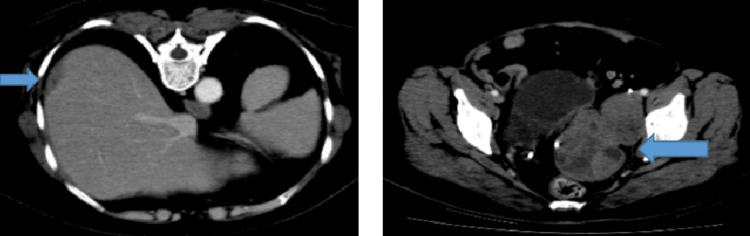
CT scan axial views showing progressive disease in terms of an increase in the size of bilateral adnexal masses and extracapsular hepatic metastasis.

The patient refused chemotherapy this time, and she was treated with hormonal therapy (letrozole) till December 2018, when her disease progressed again with an increase in the size of adnexal masses (Figure 10). She received weekly paclitaxel for 12 cycles and stopped further treatment due to recurrent infections and inpatient admissions affecting her quality of life, and progression of disease on scans. She received leprolin acetate once a month from October 2019 till February 2020 and was lost to follow-up thereafter due to the COVID-19 pandemic. Follow-up scans revealed stable disease; she received tamoxifen alternating with megestrol acetate from December 2020 till February 2021 when her disease progressed again (Figure 11). This time she was given eight cycles of carboplatin/gemcitabine till November 2021. Currently, she has been taking anastrozole since March 2022 and is under follow-up with stable disease on imaging.

## Discussion

Briefly, both of our patients were relatively older in age and had early-stage disease (stage IA) on presentation. Both the patients received no adjuvant chemotherapy after surgery at diagnosis and they were offered active surveillance due to no residual disease and negative margins with intact capsules. Both of the patients relapsed multiple times and were managed with debulking surgeries, chemotherapy, and hormonal therapy throughout their course of treatment.

GCT is common in postmenopausal women with common clinical manifestations of abdominal pain, pelvic mass, and vaginal bleeding [7]. Better overall survival and disease-free survival outcomes have been shown in patients with GCTs who presented at an early stage, underwent optimal debulking, and had no residual disease on postoperative imaging [8,9]. According to Al-Badawi et al., residual disease following surgery is one of the factors affecting the ability of individuals with GCTs to live without developing a disease progression [10]. Patients with completely resected stage I disease (with no residual disease) have a five-year survival of around 90% [11-13]. In addition, Bin Naeem et al. reported postmenopausal status, advanced disease at presentation, capsular rupture, the presence of ascites, omental involvement, peritoneal dissemination, and residual disease after surgical resection affected disease-free survival adversely [14].

Though variable combinations of postoperative care, including chemotherapy, radiation, and hormonal agents have been suggested; yet there are no set standards and the benefit is unclear [15-17]. There are some reports that suggest the role of adjuvant chemotherapy for every patient with stage IC disease and higher; others, however, recommend adjuvant chemotherapy for such patients aged 40 years or above [18]. Still, others recommend adjuvant chemotherapy for patients with residual disease after surgery or altogether when there is a recurrence of the disease. Optimal debulking surgery and adjuvant chemotherapy remain the most effective treatment as per the 2018 ESMO guidelines [19].

There is a growing interest in the long-term prognosis of individuals with stage 4 recurrent granulosa cell ovarian tumors. Though rare and frequently indolent compared to epithelial ovarian cancers, granulosa cell ovarian tumors pose significant challenges when there is a recurrence with advanced disease (stage IV). Surgery with optimal debulking remains the standard treatment for recurrences, if feasible [18]. Chemotherapy is an option for patients with recurrent disease who underwent resection with no residual disease (adjuvant) or for those who have unresectable disease or are not candidates for surgery [18]. Drugs like paclitaxel and platinum-based agents are used in chemotherapy regimens because of their effectiveness in slowing the course of illness [19]. Typically, paclitaxel and carboplatin (PC) will be the choice of chemotherapy at recurrence in case BEP was given previously and vice versa and BEP is best avoided in patients >40 years of age [20-22]. Hormonal agents like tamoxifen, progesterone, or a combination of both can sometimes result in long-term responses [23,24]. This is partly determined by the expression of FLOX2 mutation, which leads to aromatization and production of estrogen in access, and estrogen receptor beta and progesterone receptor are the main carriers of the pathway of aromatization [25]. van Meurs et al. have reported a 71% objective response rate with hormone therapy, reaching up to 100% in women who received aromatase inhibitors alone or in combination, with a higher response rate in ER (estrogen receptor) negative and PR (progesterone receptor) positive and disease progression in ER/PR negative patients [24]. Regarding targeted therapies, phase 2 studies of bevacizumab have shown encouraging results with an overall response rate of 94.5%, which is based on increased expression of vascular endothelial growth factor (VEGF) levels in serum, with an autocrine effect on the tumor [26,27].

In summary, both of our patients had stage I disease at presentation and underwent optimal debulking with no residual disease and with no adjuvant chemotherapy, with an average recurrence interval of five years (seven years and three years, respectively), which is consistent with four to six years recurrence interval reported in the literature [28]. Despite multiple recurrences, both patients survived for more than 20 years on multiple therapies so far. This confirms the notion that early-stage disease at presentation and optimal debulking with no residual disease prove to be factors favoring long-term outcomes.

## Conclusions

Adult GCT is a disease of older age, postmenopausal women with a better prognosis when they present with early-stage disease and undergo optimal debulking surgery alone with no residual disease. Recurrences of the disease can also be treated with surgery to achieve no residual disease followed by adjuvant chemotherapy. Further recurrences can be treated with surgery, chemotherapy, hormonal therapy, and targeted therapy with favorable outcomes.
